# A Glimpse of the genomic diversity of haloarchaeal tailed viruses

**DOI:** 10.3389/fmicb.2014.00084

**Published:** 2014-03-12

**Authors:** Ana Senčilo, Elina Roine

**Affiliations:** Department of Biosciences and Institute of Biotechnology, University of HelsinkiHelsinki, Finland

**Keywords:** archaea, haloarchaea, tailed virus, genome, bacteriophage

## Abstract

Tailed viruses are the most common isolates infecting prokaryotic hosts residing in hypersaline environments. Archaeal tailed viruses represent only a small portion of all characterized tailed viruses of prokaryotes. But even this small dataset revealed that archaeal tailed viruses have many similarities to their counterparts infecting bacteria, the bacteriophages. Shared functional homologs and similar genome organizations suggested that all microbial tailed viruses have common virion architectural and assembly principles. Recent structural studies have provided evidence justifying this thereby grouping archaeal and bacterial tailed viruses into a single lineage. Currently there are 17 haloarchaeal tailed viruses with entirely sequenced genomes. Nine viruses have at least one close relative among the 17 viruses and, according to the similarities, can be divided into three groups. Two other viruses share some homologs and therefore are distantly related, whereas the rest of the viruses are rather divergent (or singletons). Comparative genomics analysis of these viruses offers a glimpse into the genetic diversity and structure of haloarchaeal tailed virus communities.

Viruses infecting haloarchaea come in a variety of virion morphotypes: spindle-shaped, pleomorphic, icosahedral and head-and-tail (or tailed) (Roine and Oksanen, 2011; Atanasova et al., 2012; Pietilä et al., 2013a). Yet, tailed viruses comprise the majority of the studied viruses infecting haloarchaea (Table 1). Despite the many early studies on ϕH genome and its rearrangements (Reiter et al., 1988) as well as detailed studies on ϕCh1 virus (Witte et al., 1997; Baranyi et al., 2000; Klein et al., 2002; Rössler et al., 2004) we have had relatively little in-depth information about the haloarchaeal tailed virus genomes until recently (Klein et al., 2012; Pietilä et al., 2013b,c; Senčilo et al., 2013). The situation changed partly due to the recent technological advancements that have made for instance the sequencing of viral genomes much cheaper and faster than before. This caused an exponential increase in the number of sequencing projects focusing on separate virus genomes or on metaviromes from hypersaline environments (Santos et al., 2010; Boujelben et al., 2012; Garcia-Heredia et al., 2012; Pietilä et al., 2013b,c; Senčilo et al., 2013). While metaviromes revealed the richness and diversity of the viral communities present in hypersaline environments, whole-genome sequencing of isolated viruses provided more complete genomic information embedded in a clear biological context. The aim of this review is to summarize the findings on the 13 new complete haloarchaeal tailed virus genomes that were published in three separate papers (Pietilä et al., 2013b,c; Senčilo et al., 2013) and to combine these data with the previous knowledge of the complete genomes of haloarchaeal tailed viruses.

**Table 1:** **Characteristics of haloarchaeal tailed viruses with completely sequenced genomes**.

**Morphology**	**Subgroup (if applicable)**	**Virus**	**Origin**	**Isolation host**	**Genome type**	**Genome size (bp)**	**Nr of ORFs**	**Nr of tRNAs**	**References**
myo	HF2-like	HF1	Cheetham saltworks, Australia	*Hfx. lucentense*	Linear	75,898	112	5	Nuttall and Dyall-Smith, 1993; Tang et al., 2004
					dsDNA, DTR (306 bp)				
		HF2	Cheetham saltworks, Australia	*Hrr. coriense*	Linear	77,670	116	5	Nuttall and Dyall-Smith, 1993; Tang et al., 2002
					dsDNA, DTR (306 bp)				
		HRTV-5	Margherita di Savoia, Italy	*Hrr.* sp. s5a-3	Linear	76,134	118	4	Atanasova et al., 2012; Senčilo et al., 2013
					dsDNA, DTR (271 bp)				
		HRTV-8	Samut Sakhon, Thailand	*Hrr.* sp. B2-2	Linear	74,519	124	4	Atanasova et al., 2012; Senčilo et al., 2013
					dsDNA, DTR (346 bp)				
	HRTV-7 like	HRTV-7	Margherita di Savoia, Italy	*Hrr.* sp. B2-2	linear	69,048	105	1	Atanasova et al., 2012; Senčilo et al., 2013
					dsDNA, DTR (340 bp)				
		HSTV-2	Eilat, Israel	*Hrr. sodomense*	Linear	68,187	103	1	Atanasova et al., 2012; Pietilä et al., 2013b
					dsDNA, DTR (340 bp)				
		φCH1	Spontaneous release from *Nab magadii*	*Nab. magadii* (L13)	circ. perm. dsDNA	58,498 (+80-700 nt RNA)	98	−	Witte et al., 1997; Klein et al., 2002
		HGTV-1	Samut Sakhon, Thailand	*Hgn.* sp. SS5-1	circ. perm. dsDNA	143,855	281	36	Atanasova et al., 2012; Senčilo et al., 2013
sipho	HCTV-1 like	HCTV-1	Margherita di Savoia, Italy	*Har. californiae*	Linear	103,257	160	1	Kukkaro and Bamford, 2009; Senčilo et al., 2013
					dsDNA, DTR (739 bp)				
		HCTV-5	Samut Sakhon, Thailand	*Har. californiae*	Linear	102,105	166	1	Atanasova et al., 2012; Senčilo et al., 2013
					dsDNA, DTR (583 bp)				
		HVTV-1	Samut Sakhon, Thailand	*Har. vallismortis*	Linear	101,734	173	1	Atanasova et al., 2012; Pietilä et al., 2013b
					dsDNA, DTR (585 bp)				
		HCTV-2	Samut Sakhon, Thailand	*Har. californiae*	circ. perm. dsDNA	54,291	86	−	Atanasova et al., 2012; Senčilo et al., 2013
		HHTV-2	Samut Sakhon, Thailand	*Har. hispanica*	circ. perm. dsDNA	52,643	88	−	Atanasova et al., 2012; Senčilo et al., 2013
		BJ1	Lake Bagaejinnor, China	*Hrr.* sp. BJ1 B11	Linear	42,271	70	1	Pagaling et al., 2007
					dsDNA^*^				
		HHTV-1	Margherita di Savoia, Italy	*Har. hispanica*	circ. perm. dsDNA	49,107	74	−	Kukkaro and Bamford, 2009; Senčilo et al., 2013
		HRTV-4	Margherita di Savoia, Italy	*Hrr.* sp. s5a-3	circ. perm. dsDNA	35,722	73	−	Atanasova et al., 2012; Senčilo et al., 2013
podo		HSTV-1	Margherita di Savoia, Italy	*Har. sinaiiensis*	circ. perm. dsDNA	32,189	53	−	Atanasova et al., 2012; Pietilä et al., 2013c

* The genome type of BJ1 has not been clearly established; Hrr, Halorubrum; Nab, Natrialba; Hfx, Haloferax; Hgn, Halogranum; circ. perm., circularly permuted; DTR, direct terminal repeats; myo, myovirus; sipho, siphovirus; podo, podovirus.

## Classification of prokaryotic tailed viruses

Tailed euryarchaeal (including haloarchaeal) viruses have been shown to have many properties in common with their bacterial counterparts, the bacteriophages, starting from the morphology and the genome structure to gene regulation and some protein homologs (Torsvik and Dundas, 1974; Stolt and Zillig, 1994; Porter et al., 2007). Tailed bacteriophages are classified into order *Caudovirales*, which is further divided into three families according to the tail morphology: *Myoviridae* characterized by long contractile tails, *Siphoviridae* (long, non-contractile, but flexible tails) and *Podoviridae* (short non-contractile tails) (King et al., 2012). Some of the haloarchaeal tailed viruses have also been classified according to the criteria of the International Committee on Taxonomy of Viruses (ICTV) (King et al., 2012). The genus “PhiH-like viruses” belongs to the family *Myoviridae* and contains the species *Halobacterium* phage ϕH and a candidate *Halobacterium* phage Hs1 (King et al., 2012). Also HF2 has been added as a putative member of the *Myoviridae* family (King et al., 2012).

Before the times of having the means to generate massive amounts of sequence data, viral classification mainly based on virion morphology, the genome type (circular or linear ss/dsDNA or RNA) and host range, seemed rather straightforward. The current ease of genome sequencing revealed the Pandora's box of the prokaryotic virus genomes. First of all, at the nucleotide sequence level the genomes are often very different from each other with no sequence similarity at all. In addition, mosaicism, the inherent feature of the prokaryotic viral genomes (Hendrix et al., 1999; Juhala et al., 2000; Lawrence et al., 2002; Krupovič et al., 2011), raises serious questions about the criteria to be used in classification. It has been proposed that in the absence of nucleotide or amino acid sequence similarity, the higher order classification of viruses should be based on the virion morphology and the major capsid protein fold (MCP) (Bamford et al., 2002, 2005; Abrescia et al., 2012). Viruses having the same MCP fold could then be grouped into lineages, and tailed bacteriophages were suggested to belong to the so called Hong Kong 97 (HK97)-like lineage together with the herpesviruses (Bamford, 2003; Bamford et al., 2005; Abrescia et al., 2012). Recent structural studies on haloarchaeal podovirus HSTV-1 suggested that it also has the HK97 MCP fold thereby justifying the placement of archaeal tailed viruses into HK97-like lineage (Pietilä et al., 2013c).

## Characteristics of haloarchaeal tailed virus genomes

At the moment there are 43 haloarchaeal tailed viruses reported (Kukkaro and Bamford, 2009; Atanasova et al., 2012; Sabet, 2012) and 17 completely sequenced genomes comprise approximately 1.2 Mb of sequence information (Klein et al., 2002; Tang et al., 2002, 2004; Pagaling et al., 2007; Pietilä et al., 2013b,c; Senčilo et al., 2013). Also approximately 58 kb of the ϕH genome has been sequenced (Porter et al., 2007). In addition to that, several proviral regions found in haloarchaeal genomes extend our knowledge of the gene pool of haloarchaeal tailed viruses (Krupovič et al., 2010; Senčilo et al., 2013). Complete genomes of haloarchaeal tailed viruses range from approximately 32 to 144 kb in size (Table 1). Similarly to tailed bacteriophages, the genomes of haloarchaeal tailed viruses are either circularly permuted or non-permuted dsDNA molecules with direct terminal repeats (Klein et al., 2002; Tang et al., 2002, 2004; Pagaling et al., 2007; Pietilä et al., 2013b,c; Senčilo et al., 2013). The genomes have rather high GC percentage (above 50% on average), which is also characteristic of haloarchaea (Klein et al., 2002; Tang et al., 2002, 2004; Oren, 2006; Pagaling et al., 2007; Pietilä et al., 2013b,c; Senčilo et al., 2013). Similar GC percentages suggest that the viruses are well-adapted to the codon usage of their hosts.

Annotation of the haloarchaeal tailed virus genomes is very often based on the similarity to bacteriophage genes (Klein et al., 2002; Tang et al., 2002, 2004; Pagaling et al., 2007; Krupovič et al., 2011; Pietilä et al., 2013b,c; Senčilo et al., 2013). Indeed, haloarchaeal tailed viruses share many similarities with bacteriophages both in terms of genome content and organization (Krupovič et al., 2011). In general, however, putative function can be assigned to no more than 20% of the new haloarchaeal tailed virus genes (Pagaling et al., 2007; Pietilä et al., 2013b,c; Senčilo et al., 2013). Large terminase subunit is among the most conserved proteins of prokaryotic tailed viruses and it was annotated in all haloarchaeal tailed virus genomes described to date (Klein et al., 2002; Tang et al., 2002, 2004; Pagaling et al., 2007; Pietilä et al., 2013b,c; Senčilo et al., 2013).

**Figure 1:**
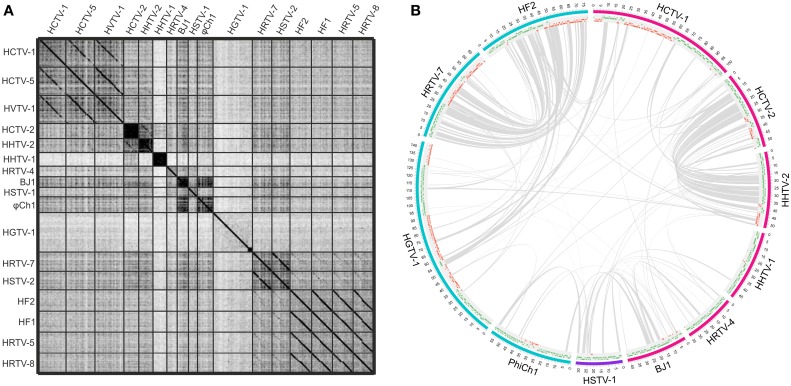
**Genomic comparisons of the haloarchaeal tailed viruses with completely sequenced genomes. (A)** Dotplot alignment of the genomes. Reverse complements of HF1 and HF2 genome sequences were used for the analyses in order to conform to the structure of the other haloarchaeal tailed virus genomes. The image was generated using the Gepard software (Krumsiek et al., 2007). **(B)** Circular visualization of the homologous proteins shared between the selected virus representatives from each of the delineated groups and singletons. The outermost track represents the genome maps with the coordinates (kbp). The myoviral genomes are marked in blue, siphoviral in pink and podoviral in violet. The following track displays the annotated ORFs (marked in green and red on the positive and the negative strands, respectively). Gray lines link pairs of genes coding for the putative homologs. Here proteins are defined as homologous if they share over 30% amino acid identity when aligned with EMBOSS Needle tool (Needleman and Wunsch, 1970). The image was generated using Circos software (Krzywinski et al., 2009).

While the genomes of some haloarchaeal tailed viruses are collinear and highly similar at the nucleotide level, other viruses share up to several distant protein homologs at most (Figure 1). None of the completely sequenced genomes displayed close similarity to the putative proviral regions identified in the haloarchaeal genomes (Krupovič et al., 2010; Senčilo et al., 2013). Among the 17 haloarchaeal tailed viruses, three groups of closely related viruses can be delineated based on the nucleotide sequence alignments (Figure 1A). Here we name these groups according to the first described representative: HF2-like, HRTV-7-like and HCTV-1-like groups (Nuttall and Dyall-Smith, 1993; Atanasova et al., 2012; Senčilo et al., 2013).

### HF2-like viruses

The biggest group is HF2-like myovirus group, which, besides HF2, includes HF1, HRTV-5, and HRTV-8 viruses (Figure 1A) (Nuttall and Dyall-Smith, 1993; Atanasova et al., 2012; Senčilo et al., 2013). HF2-like viruses originate from spatially and temporally different environmental samplings (Nuttall and Dyall-Smith, 1993; Atanasova et al., 2012). Nevertheless, viruses share extensive similarity at the nucleotide level and subsequently most of their encoded proteins are homologous (Tang et al., 2002, 2004; Senčilo et al., 2013). Highly similar genomic regions are interrupted by non-homologous regions suggestive of the mosaic nature of HF2-like virus genomes (Tang et al., 2002, 2004; Senčilo et al., 2013). The clearest example is provided by HF1 and HF2 virus genomes, which are almost identical over 48 kb followed by a more diverged 28 kb region (Tang et al., 2004). The divergent region, among other putative proteins, codes for the tail fiber protein, which may be responsible for different host specificities of these two viruses (Tang et al., 2004). Majority of the non-conserved proteins in HF2-like viruses have no predicted function with an exception of putative restriction endonuclease and methylase (HF2p074 gene in HF2) found in all viruses except for HRTV-8, and HNH endonuclease found only in HRTV-8 (gene 43) (Tang et al., 2002, 2004; Senčilo et al., 2013).

### HRTV-7-like viruses

HF2-like viruses share some similarities with HRTV-7-like myoviruses, HRTV-7 and HSTV-2 (Figures 1A,B) (Pietilä et al., 2013b; Senčilo et al., 2013). Homologous genome regions are mostly located in the gene cluster coding for structural and assembly proteins (Pietilä et al., 2013b; Senčilo et al., 2013). Cryo-electron microscopy studies on HSTV-2 virus revealed that its capsid has a *T* = 7 symmetry (Pietilä et al., 2013b). However, known viruses having capsids with this T-number, such as P22, package smaller genomes than that of HSTV-2 (Parent et al., 2010; Pietilä et al., 2013b). Therefore it was suggested that HSTV-2 capsids accommodate minor proteins, which increase the capsid volume (Pietilä et al., 2013b). Since all HRTV-7-like and HF2-like viruses have homologous MCPs as well as hypothetical proteins suggested to act as minor capsid proteins, it is likely that the capsid structures of all these viruses are similar (Pietilä et al., 2013b).

### HCTV-1-like and other related siphoviruses

HCTV-1, HCTV-5, and HVTV-1 viruses encompass the HCTV-1-like virus group and are the only closely related haloarchaeal siphoviruses described to date (Figure 1A) (Pietilä et al., 2013b; Senčilo et al., 2013). HVTV-1 and HCTV-5 show similarity throughout their genomes, whereas HCTV-1 has a diverged genome region coding for tail structural and assembly proteins (Pietilä et al., 2013b; Senčilo et al., 2013). Another notable difference is rather high abundance of homing endonuclease genes in HVTV-1 and HCTV-5 genomes compared to HCTV-1 (Pietilä et al., 2013b; Senčilo et al., 2013). Structural studies available only for HVTV-1 virus showed that its capsomers are arranged in a *T* = 13 lattice (Pietilä et al., 2013b).

Siphoviruses HCTV-2 and HHTV-2 also show some similarity to each other at the nucleotide sequence level and share a number of protein homologs (Figures 1A,B) (Senčilo et al., 2013). As is the case for HF2-like and HRTV-7-like groups of viruses, similarities among HCTV-2 and HHTV-2 are mostly concentrated within the cluster of head and tail structural and assembly proteins (Figure 1B) (Senčilo et al., 2013).

### Singletons

Siphovirus HHTV-1 is the most divergent among the completely sequenced haloarchaeal tailed viruses (Senčilo et al., 2013). The only homolog it shares with other haloarchaeal tailed viruses is a putative PCNA, which is similar to HSTV-1 podoviral PCNA (Figure 1B). Other two siphoviruses having no close relatives among and the entirely sequenced haloarchaeal tailed viruses are HRTV-4 and BJ1 (Pagaling et al., 2007; Senčilo et al., 2013). However, even in these four diverged siphoviruses some of the structural and assembly proteins as well as putative proteins involved in nucleic acid metabolism were annotated based on the similarities to their counterparts in bacteriophages (Pagaling et al., 2007; Senčilo et al., 2013). The genome of the siphovirus HRTV-4 (Senčilo et al., 2013) shows close relatedness to an environmental clone eHP-10 (Garcia-Heredia et al., 2012). The two sequences align along approximately half of the length with close to 80% nucleotide sequence identity.

Although ϕCh1 is rather distinct from other fully sequenced haloarchaeal tailed viruses, it is one of the best characterized haloarchaeal viruses to date (Witte et al., 1997; Klein et al., 2002, 2012). ϕCh1 is a temperate virus infecting *Natrialba (Nab.) magadii* cells (Witte et al., 1997). The most unusual feature of the ϕCh1 virus is that its particles along with the genomic dsDNA contain 80–700 nt RNA molecules of host origin (Witte et al., 1997). A 12 kb region of ϕCh1 genome is highly similar to the φH virus L-fragment (Gropp et al., 1992; Klein et al., 2002). This fragment of φH virus was shown to be capable of autonomous replication in a plasmid state (pφHL) (Gropp et al., 1992). It contains genes coding for proteins involved in replication, plasmid stabilization and gene expression regulation (Gropp et al., 1992).

The ϕCh1 genome region and pφHL align along almost the whole length with an exception of 1.7 kb fragment, which is in the inverse orientations in the two (Klein et al., 2002). Direct repeats flanking the fragment suggested that the rearrangement was a result of recombination between these repeats (Klein et al., 2002). ϕCh1 genome contains a number of inverted repeats, one pair of which is involved in a phase variation system (Rössler et al., 2004; Klein et al., 2012). This system results in the production of two different variants of ϕCh1 tail fiber protein (Klein et al., 2012).

HGTV-1 myovirus currently holds the record for having the largest genome among all described archaeal viruses (Senčilo et al., 2013). The genome of this virus has at least two distinctive features. First, it encodes unusually high number of tRNAs (36 in total) for all universal amino acids (Senčilo et al., 2013). Second, majority of ORFs located in HGTV-1 left-hand side of the genome are preceded by a conserved DNA motif, containing TATA box-like region and an inverted repeat (Senčilo et al., 2013). Similarity of these structures to promoter stem loops (PesLSs) of T4-type bacteriophages led to the suggestion that as in T4-like bacteriophages, these DNA motifs in HGTV-1 are responsible for transcription regulation and genome shuffling (Arbiol et al., 2010; Senčilo et al., 2013). Therefore, the mechanism of generating genetic diversity may also be shared among bacterial and archaeal tailed viruses in addition to the already pronounced similarity of structural and assembly proteins (Senčilo et al., 2013).

To date, HSTV-1 is the only reported archaeal podovirus (Pietilä et al., 2013c). It is also the only archaeal tailed virus for which the MCP fold was determined (Pietilä et al., 2013c). Despite its podoviral morphotype, HSTV-1 shares a handful of homologs with haloarchaeal myo- and siphoviruses (Figure 1B). These include the MCM DNA helicase, terminase large subunit, PCNA as well as several hypothetical proteins (Figure 1B).

## Conclusion

The growing number of complete genomes of haloarchaeal tailed viruses allowed us to determine groups of related viruses with more than two members. As new sequences are added, the groups are increasing in size and number. In addition to that, new singletons appear. A similar trend was also noticed for the growing database of complete mycobacteriophage genomes (Hatfull, 2012). The 17 completely sequenced haloarchaeal tailed viruses can be currently divided into 3 groups of closely related viruses, a pair of more distantly related siphoviruses and 6 singletons. Comparative genomics analysis of these genomes further corroborated several observations made earlier. First, different levels of relatedness can be observed among the haloarchaeal tailed virus genomes. In general this relatedness correlates neither with the place nor with the time of sampling for the virus isolation. For example very closely related viruses such as HF2-like viruses, were isolated from geographically distant sources in the span of almost 20 years (Nuttall and Dyall-Smith, 1993; Atanasova et al., 2012). Second, virion structure and assembly proteins are generally more conserved among the viruses, as is apparent from the examples of HF2-like and HRTV-7-like groups of viruses as well as HCTV-2 and HHTV-2 viruses (Pietilä et al., 2013b; Senčilo et al., 2013). Finally, the analysis of the extended data set did not yield more information on some single divergent viruses such as HHTV-1. This case examplifies the gaps in our knowledge and highlights the fact that more sequences are needed for the deeper understanding of genetic diversity and structure of the viral communities as well as evolutionary processes shaping them.

### Conflict of interest statement

The authors declare that the research was conducted in the absence of any commercial or financial relationships that could be construed as a potential conflict of interest.
